# Building a tobacco user registry by extracting multiple smoking behaviors from clinical notes

**DOI:** 10.1186/s12911-019-0863-3

**Published:** 2019-07-25

**Authors:** Ellen L. Palmer, Saeed Hassanpour, John Higgins, Jennifer A. Doherty, Tracy Onega

**Affiliations:** 1 Dartmouth College, HB 7922, 03755 Hanover, NH USA; 2 Dartmouth College, HB 7261, 03755 Hanover, NH USA; 3 Dartmouth College, HB 7920, 03755 Hanover, NH USA; 4 Huntsman Cancer Institute, University of Utah, 2000 Circle of Hope Dr, Salt Lake City, UT 84112 USA; 5 Dartmouth College, HB 7927, 03755 Hanover, NH USA

**Keywords:** Smokers registry, Informatics pipeline, Electronic health records, Natural language processing

## Abstract

**Background:**

Usage of structured fields in Electronic Health Records (EHRs) to ascertain smoking history is important but fails in capturing the nuances of smoking behaviors. Knowledge of smoking behaviors, such as pack year history and most recent cessation date, allows care providers to select the best care plan for patients at risk of smoking attributable diseases.

**Methods:**

We developed and evaluated a health informatics pipeline for identifying complete smoking history from clinical notes in EHRs. We utilized 758 patient-visit notes (from visits between 03/28/2016 and 04/04/2016) from our local EHR in addition to a public dataset of 502 clinical notes from the 2006 i2b2 Challenge to assess the performance of this pipeline. We used a machine-learning classifier to extract smoking status and a comprehensive set of text processing regular expressions to extract pack years and cessation date information from these clinical notes.

**Results:**

We identified smoking status with an F1 score of 0.90 on both the i2b2 and local data sets. Regular expression identification of pack year history in the local test set was 91.7% sensitive and 95.2% specific, but due to variable context the pack year extraction was incomplete in 25% of cases, extracting packs per day or years smoked only. Regular expression identification of cessation date was 63.2% sensitive and 94.6% specific.

**Conclusions:**

Our work indicates that the development of an EHR-based Smokers’ Registry containing information relating to smoking behaviors, not just status, from free-text clinical notes using an informatics pipeline is feasible. This pipeline is capable of functioning in external EHRs, reducing the amount of time and money needed at the institute-level to create a Smokers’ Registry for improved identification of patient risk and eligibility for preventative and early detection services.

**Electronic supplementary material:**

The online version of this article (10.1186/s12911-019-0863-3) contains supplementary material, which is available to authorized users.

## Background

Smoking remains the leading cause of preventable disease and death in the US [1], with approximately 20% of all deaths in the US attributable to smoking [2–4]. However, despite successful public health efforts to reduce the prevalence of cigarette smoking over the past several decades, the Behavioral Risk Factor Surveillance System (BRFSS) questionnaire found that 17.5% of US adults are still current smokers and 25.3% are former smokers in 2016 [5]. Encouragingly, the majority of current smokers plan to quit [6], and are more likely to successfully do so with clinician-delivered cessation assistance [7], However, in order for clinicians to appropriately deliver smoking cessation assistance and appropriate screening for smoking attributable diseases, smokers need to be identified and tracked, typically within a hospital’s electronic health records (EHR) software.

The Centers for Medicare and Medicaid Services (CMS) initially required health care providers, as part of the Stage 2 Meaningful Use Objectives, to collect smoking status information within structured fields in their EHRs on at least 80% of all patients over the age of 13 to remain eligible for reimbursement, though this requirement has been removed due to a high level of compliance [8]. However, there are no requirements for collecting additional tobacco use information such as pack years, number of quit attempts, and, in former smokers, cessation dates. This additional information is necessary for taking clinically actionable steps including referring individuals to appropriate cessation intervention or screening, such as lung cancer screening [9, 10].

For nearly a decade, health informatics researchers have demonstrated that Natural language processing (NLP) techniques are a feasible way to extract smoking status from medical records [11, 12]. These algorithms effectively collect smoking status, but to date no algorithms have been published that capture other smoking behavior information such as pack years or cessation date [11, 12]. Additionally, a limitation of these smoking status algorithms is their tendency to only perform optimally in the EHRs where they are trained, limiting the transferability of such data ascertainment tools [11, 12]. Here, we: 1) present a generalizable smoking status (ever/never) algorithm which performs consistently when trained using notes from a single EHR and tested in a set of external notes; 2) develop novel algorithms for collecting tobacco cessation data and partial or complete pack year information (cigarettes per day, packs per day, number of years smoked, and pack years); and 3) evaluate clinical perception of a Smokers’ Registry design based on the information collected. We present an informatics pipeline that combines the information captured by these algorithms and can be applied to EHR to create a Smokers’ Registry. After further validation, integration of this pipeline into EHR systems could provide clinicians the information needed to more meaningfully address current and future health concerns related to patient smoking behaviors.

## Methods

In 2006, Informatics for Integrating Biology and the Bedside (i2b2) developed and published a deidentified data set which includes visit notes for 502 individuals classified as having one of five smoking status designations (never, past, current, smoker temporality unknown, and unknown smoking status) for training and testing smoking status algorithms [12]. Details on the number of notes in each class can be found in Table 1.

**Table 1:** Summary of Testing and Training Data Available for Algorithm Development

Smoking status^1^	i2b2		Local EHR					
	Smoking Status		Smoking Status		Pack years		Cessation Date	
	Train *n* = 398	Test *n* = 104	Train *N* = 533	Test *N* = 223	Train *N* = 84	Test *N* = 36	Train *N* = 54	Test *N* = 19
Never	66	16	117	51	–	–	–	–
Ever	80	25	139	64	84	26	54	19
Former	36	11	71	30	38	12	54	19
Current	35	11	58	31	39	23	–	–
Smoker	9	3	10	3	7	1	–	–
Unknown	252	63	277	108	–	–	–	–

Distribution of annotations for smoking status, pack years, and cessation date for the training and testing data from the i2b2 Challenge and our local EHR. Smoking status was determined by a manual review, with notes classified as: Never smoker, former smoker, current smoker, smoker temporality unknown (referred to as smoker), or no smoking status information (referred to as unknown). For the local EHR pack year and cessation date counts, we indicate the number of notes for which this information was identified by manual review

**Fig. 1:**
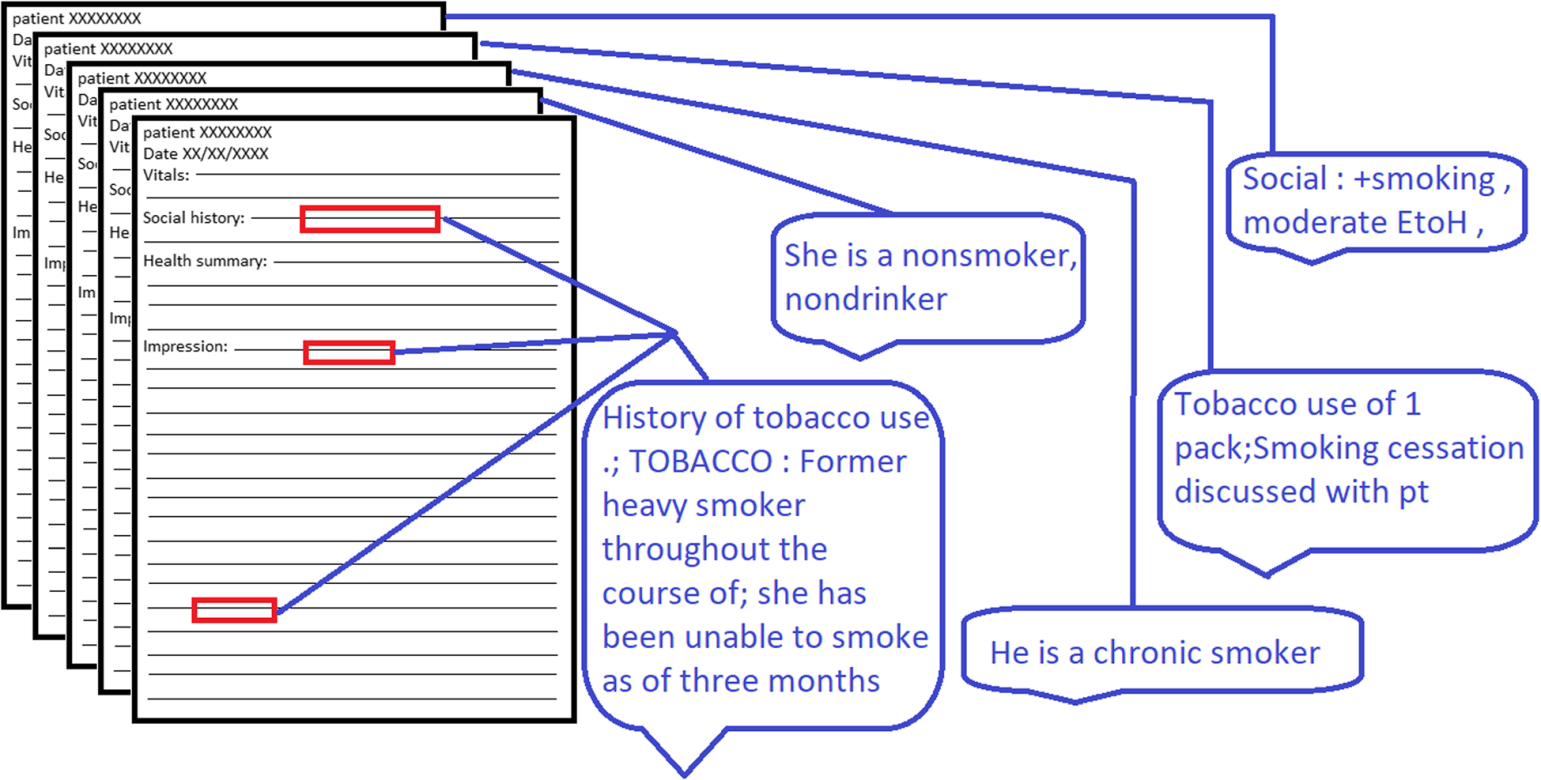
Visualization of information extraction for smoking status classification. Visualization of the process used by the smoking status algorithm. Notes were manually annotated for smoking behavior concepts in 756 local records. Many of these notes were semi-structured and contained clearly defined sections. Smoking behaviors were most often found in the “social history” and impression sections, followed by the “impressions” and “health summary” sections

To evaluate the feasibility of creating a Smoker’s Registry within our local EHR, data were also collected from 758 patient visit notes from the Dartmouth-Hitchcock healthcare system (henceforth referred to as “local”). Visit notes were extracted from the local Epic data warehouse in April of 2016. We performed an SQL keyword search on notes generated between 03/28/2016 and 04/04/2016 for patients aged 18 years or older. To ensure we had enough notes with smoking-related concepts, half of the local notes in our dataset were selected because they contained the word-stem “smok”, while the other half were pulled as a random sample of eligible notes generated from visits in the same date range. Many of the notes were semi-structured (Fig. 1), containing sections such as “vitals”, “social history”, “health summary”, and “impressions”; smoking behaviors information was most often found under “social history” or “impressions”. All participants provided written consent. This study was approved by the Dartmouth College Committee for the Protection of Human Subjects.

All smoking information in the local dataset were manually annotated by the study team between 5/1/16 and 6/30/16 for smoking status, cessation date, consumption information (including packs per day, cigarettes per day, and pack years), along with other smoking-related themes including smoking attributable diseases and quit attempts. Smoking status classification was completed using the same five classes used by i2b2: current smoker, former smoker, never smoker, smoker temporality unknown (the note indicated that the person has a smoking history, but the annotator was unable to definitively classify the patient as a current or former smoker), or no smoking status information present (labeled unknown). Manual annotation was also used to indicate which notes contained information relating to pack year history, and cessation date. The number of notes containing annotator-identified concepts related to these three concepts can be found in Table 1; annotations were completed utilizing the eHost annotation tool. [13] All data preprocessing and algorithm development were completed using Python 2.7.12. [14] Support vector machine and machine learning models were constructed using functions from the sklearn package. [15] Regular expression methods utilized functions from the re package [16]. Finally, Multi-date format recognition was completed using the datetime package [17].

Our NLP approach used a hotspot-guided support vector machine (SVM), as previously described by Cohen et al. [11]. Briefly, Cohen’s method used text within +/− 100 characters of six domain-expert identified word stems (hotspots), with notes containing no hotspots being automatically labeled “unknown”. These hotspots were “smok”, “cig”, “tobac”, “packs”, “tob”, and “nicotine”.

We assessed adaptations to Cohen’s algorithm which included evaluating the number and composition of hotspots needed and the size of the text window around hotspots by using 5-fold cross-validation on the i2b2 training set. We also tested basing the window on words instead of characters to better capture context. We assessed whole sentences and window sizes of +/− three to seven words around the hotspots, provided the words occurred within the same sentence. Both the character and word window sizes were chosen due to English modifiers most often being located close to the modified word (i.e. ‘former smoker’ is more common than ‘formerly, this patient participated in recreational smoking’), and nearly always within the same sentence.

For each of the word and character windows tested, we created the vector of features using the nltk package’s tokenize function. We removed negation words “neither”, “never”, “no”, “nor”, “not” from the standard stop words list to allow their usage in classification. Our SVM approach utilized built in functions from the sklearn package in python. We tried a variety of machine learning approaches, completing 5-fold cross-validation within the training sets of both the i2b2 and local sets. The methods tested included multi-class SVMs (varied weights and kernels), multiple logistic regression, K-nearest neighbors, gradient decent, decision trees, and random forests. We also assessed several ensembles, with each method included having an equal vote in the final classification (data not shown). When compared, we found the multi-class SVM with a linear kernel and balanced weighting performed best in the i2b2 training data and about the same as the gradient decent and ensemble approaches in our local training data. We therefore chose to only validate and present this method due to consistent performance across data sets.

Our initial work assessed SVM classifiers for identifying smoking status information. However, this approach did not allow for information to be incorporated into a structured database since it indicated if information was present but did not extract pack years or cessation dates. Therefore, we utilized regular expression methods, drawing from the patterns our study team noted while annotating notes, to create the set of rules for gathering this information. The selected regular expression stem words and patterns used to identify relevant information are in Additional file 1: Table S1. Since the i2b2 data lack annotations for these concepts, the regular expression patterns were iteratively created in the local training set and tested in the local test set once.

**Fig. 2:**
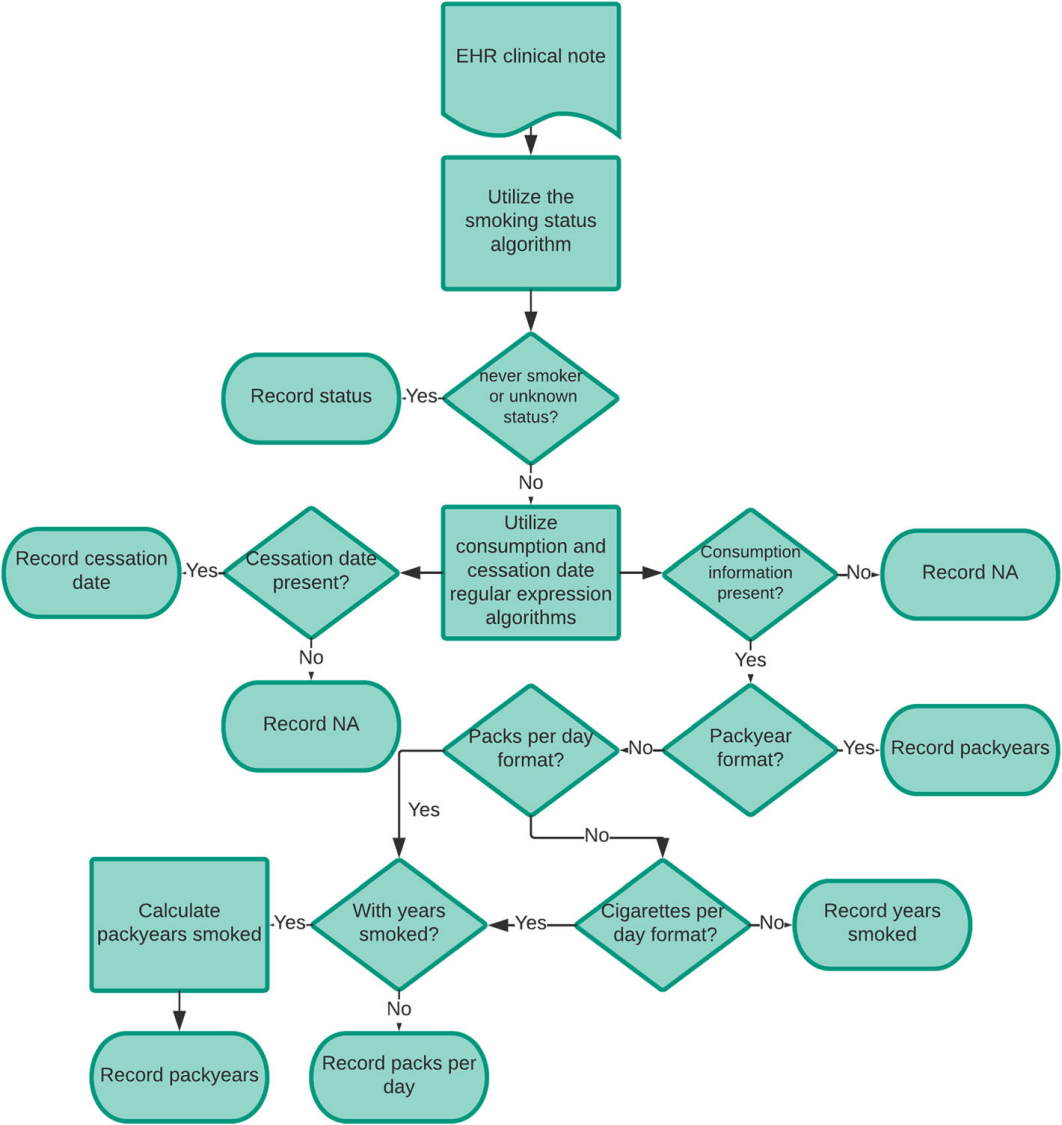
Flowchart for informatics pipeline information identification. Flowchart of the application of the pipeline to clinical notes. All notes were subjected to the smoking status algorithm. If the status assigned was never smoker or unknown, no further assessments were done. If the status assigned was current smoker, former smoker, or smoker temporality unknown, the note was assessed for pack year history and cessation date

The regular expression algorithms were only applied to records which the SVM classified as current, former, or smoker temporality unknown to reduce the risk of false positive findings of pack year history and cessation dates. This filtering step reduced the number of errors by preventing blood panels and service dates from accidentally being assessed in records classified as never smokers or unknown. If the note was classified as having a positive smoking history, the regular expressions for cessation date were applied. Since pack year history can be recorded in multiple ways, identifying pack years was done by assessing for pack years, then packs per day, then cigarettes per day, and finally years smoked. If pack years were identified, no further work was done. If partial information, such as packs per day or cigarettes per day occurred and we also found reference to years smoked, the pack year calculation was completed. In cases of partial information, such as years smoked or packs per day only, this information was collected and stored in the appropriate partial information variable. Figure 2 summarizes this workflow. The regular expression patterns utilized for identifying these various pieces of information are shown in Additional file 1: Table S2. Since the annotations generated by abstraction in the local set were for information presence, a manual review of all notes associated with extracted pack years or cessation dates was performed to verify the accuracy of the extracted pack years and cessation dates. The i2b2 data did not have annotations for these additional concepts. A manual review of only those records with pack years or cessation date found was done to confirm the accuracy of these findings.

Algorithm performance was assessed using standard informatics methods, including precision, recall, and F1-score. Precision is the quotient derived from the number of correctly assigned individuals in a category over all individuals assigned to that category, while recall is the number of correctly assigned individuals in a category over all individuals who should have been assigned to that category. The F1-score for each status category formulaically is $\frac{2\ast \left( recall\ast precision\right)}{recall+ precision}$, penalizing for too many false positive as well as too many false negatives and rewarding overall performance rather than good performance in one area. The micro-F1 score is computed using the total counts of true positives and false negatives to give a global perspective of the algorithm’s performance; Previously reported classifiers typically have micro-F1 scores of 0.85, and ideally micro-F1 scores higher than 0.9 [11, 12]. Clinically relevant metrics include sensitivity and specificity, with sensitivity having the same definition as recall and specificity being defined as the number of true negatives for a given category divided by all negatives as assessed by EP for that category.

We employed this process three times across the two data sources: Once where it was trained and tested using the i2b2 data sets, once where it was trained and tested using the local data sets, and once where we used the local training set and tested using the i2b2 testing set.

Finally, we presented the initial findings of our algorithm development, along with prototype tables of what information would be contained within the Smokers’ Registry, to several professionals who would use the proposed system (a doctor, tobacco cessation coach, and the New Hampshire Tobacco Quit Line Director). We incorporated their feedback in the design of the final set of prototype tables (Additional file 1: Tables S3-S4).

## Results

In our adaptations to the algorithms first developed by Cohen et al., we noted that only four hotspots (“smok”, “cig”, “tobac”, “nicoti”) were necessary to achieve the same level of performance they achieved with all six identified in their paper (Additional file 1: Table S1) [11]. From the annotation phase, we believed that these four hotspots were sufficient to capture most records and did not test adding new words. Further, our best model only needed +/− five words of the hotspot. This model was determined to be more sensitive than +/− three or four words and more specific than +/− six or seven words. All hot-spot windows in a given note were appended together to generate a single classification per note. Figure 1 provides a visual overview of this process.

The overall micro-F1 score when trained and tested on the i2b2 notes was 0.90. We achieved the same micro-F1 score of 0.90 when we trained and tested on the local notes. Finally, we achieved a micro-F1 score of 0.88 when we trained the smoking status algorithm on the local training set and tested with the i2b2 test set. Per class precision, recall, and F1 scores are included in Table 2. The specificity of most classes was high, with the lowest class specificity of 94% (never smoker in all 3 analyses, smoker in the locally trained i2b2 tested). The sensitivity statistic was more modest, with all three smoking categories having sensitivities at or below 73% in i2b2, at or below 84% in the local EHR notes, and at or below 64% in the locally trained and i2b2 tested analysis (Table 2). However, much of the misclassification was across smoking categories. When we combined the three smoking categories into one category (ever smoker) for the i2b2 trained and tested analysis, this combined group had a specificity of 99% and sensitivity of 96%. Similarly, in the locally trained and tested analysis, we had a specificity of 96% and sensitivity of 90% for the ever smoker category. Finally, in the locally trained and i2b2 tested analysis we had a specificity of 94% and sensitivity of 80% for the ever smoker category. Never smoker and unknown sensitivities and specificities remained unchanged in all three analyses.

**Table 2:** Summary Statistics from Smoking Status Algorithm Testing

a)	i2b2 trained and tested					
	*Micro F1: 0.90*					
	Precision	Recall	F1-Score	N notes	Sensitivity	Specificity
Never	0.94	0.94	0.94	16	94%	94%
Ever	–	–	–	25	94%	99%
Former	0.73	0.73	0.73	11	73%	97%
Current	0.62	0.73	0.67	11	73%	95%
Smoker	0.0	0.0	0.0	3	0%	99%
Unknown	1.00	1.00	1.00	63	100%	100%
b)	Local record trained and tested					
	*Micro F1: 0.90*					
	Precision	Recall	F1-Score	N notes	Sensitivity	Specificity
Never	0.83	0.98	0.90	51	98%	94%
Ever	–	–	–	64	90%	96%
Former	0.93	0.83	0.88	30	83%	99%
Current	0.79	0.84	0.81	31	84%	96%
Smoker	0.33	0.33	0.33	3	33%	99%
Unknown	0.99	0.92	0.95	108	92%	99%
c)	Local record trained, i2b2 record tested					
	*Micro F1: 0.88*					
	Precision	Recall	F1-Score	N notes	Sensitivity	Specificity
Never	0.75	0.94	0.83	16	94%	94%
Ever	–	–	–	25	80%	94%
Former	0.88	0.64	0.74	11	64%	99%
Current	0.86	0.55	0.67	11	55%	99%
Smoker	0.00	0.00	0.00	3	0%	94%
Unknown	1.00	1.00	1.00	63	100%	100%

Overall F1-score, and by smoking status precision, recall, F1-score, sensitivity, and specificity for a) i2b2 note trained and tested b) local note trained and tested and c) local note trained and i2b2 note tested data sets

The regular expression pack year algorithm was 92% sensitive and 95% specific, identifying pack year information on 33 of the 36 individuals for which we annotated the information present within the local notes (Table 3). When the extracted cigarettes per day, packs per day, and pack year numbers were manually compared to the text notes, about a quarter of them had errors in the numeric reporting. The regular expression cessation date algorithm found 12 of the 19 cessation dates and had a sensitivity of 63% and specificity of 95% (Table 3). All dates successfully extracted matched the cessation date in the notes upon manual review.

**Table 3:** Summary Statistics from the Pack Year and Cessation Date Algorithms

Annotation	a) Pack years regular expression extraction		Annotation	b) Cessation date regular expression extraction	
	Sensitivity: 91.7% Specificity: 95.2%			Sensitivity: 63.2% Specificity: 94.6%	
	Pack year history found	Pack year history not found		Cessation date found	Cessation date not found
Pack year history recorded	33	3	Cessation date recorded	12	7
No pack year history recorded	8	179	Cessation date not recorded	11	193

Sensitivity, specificity, and crosstabulation of the annotation vs. the classification of a) the pack year algorithm and b) the cessation date algorithm

In the i2b2 set, we found 4 cessation dates were correctly extracted, 5 pack year histories correctly extracted, 2 partial smoking histories correctly extracted, and 2 partial smoking histories which were incorrectly extracted. Missed extractions could not be assessed due to the lack of annotations, resulting in this assessment being limited in scope to only positive findings.

After discussing the informational strengths and limitations of these tools with our clinical collaborators, we finalized a set of tables which could be developed within an EHR system using existing patient data and the information collected from this informatics pipeline (Additional file 1: Tables S3-S4).

## Discussion

We have demonstrated that with minor adaptations to existing smoking status algorithms and the implementation of a few simple rules, the construction of a Smokers’ Registry within an EHR utilizing information found in free-text clinical notes is possible. While this pipeline is not the first proposed informatics pipeline or Smokers’ Registry [18–20], to our knowledge it is the first to successfully capture smoking status, pack year history, and quit date within one workflow. Further, we believe that the performance of our pipeline’s smoking status classifier (i.e., when trained in the local notes and tested in the i2b2 notes), demonstrates that it has the potential to be implemented in multiple EHRs with minimal local EHR retraining, though additional testing in external EHRs is needed to confirm this potential. While the pack year history and quit date algorithms could not be formally tested in the i2b2 data due to a lack of annotations, we assessed the i2b2 test set using the full pipeline and identified both pack years and quit date. Since this allowed us to assess positive findings, we were able to confirm the error rate of ~ 25% for pack year extraction. However, since these notes do not have annotations, we may have missed some notes that do contain smoking behaviors information c since we did not complete a full review of all notes, and thus cannot calculate sensitivity or specificity. Further work is needed to determine whether these algorithms are generalizable to other EHRs.

In constructing algorithms to assist in clinical decision making, both the accuracy and interpretability of the algorithms needs to be considered. Since smoking behavior collection is pertinent for all patients, not just a small subset, our process also needed to be scalable to a large hospital system. Therefore, we chose to test only computationally inexpensive algorithms that could be presented easily to a non-expert audience. Cohen’s hotspot method was complimentary to the algorithm selection process as hotspot-identified windows reduce the dimensionality of the data, using only the pertinent sections of clinical notes instead of the entire note. Further, by adapting to utilizing words instead of characters or n-mers (constructed words of n-characters long) we preserve some context and can extract the classification vector in a human-readable format – a process that allows us to show clinicians exactly what the SVM sees and uses to classify records.

There are numerous clinical and administrative benefits of capturing all information relating to smoking behaviors from the EHR. From a clinical perspective, creating a Smokers’ Registry has the potential to: 1) improve cessation service access by generating lists of current smoker patients with appointments for cessation counselors, allowing these specialists to be available for warm handoffs immediately following routine appointments [21–23]; 2) identify patients eligible for lung cancer screening and other services not relevant to the general population [24–28]; 3) increase the visibility of tobacco use and available treatment resources to clinical practitioners, which may lead to improved cessation rates and more effective preventative care [9, 28–36]; and 4) facilitate interventional research aimed at smoking related behaviors and/or diseases. Benefits from an administrative perspective include: 1) better characterization of their high-risk patient population [34, 37–39] and 2) the ability to analyze departmental needs based on these patient characteristics, which could lead to more informed decision making for resource and personnel allocation [30, 31, 34, 36–38, 40–46].

To ensure that our proposed registry design would be useful to clinicians without adding additional complexity to an already overburdened system of documentation [47], we presented the results of our algorithms and a mockup of the registry tables to a physician, a tobacco cessation counselor, several health services researchers, and the New Hampshire Tobacco Quit Line Director. Feedback about the perceived benefit of the proposed pipeline, implementation concerns, and basic data structure design needs for successful clinical integration were incorporated in to the final proposed table designs (Additional file 1: Tables S3-S4).

While our smoking status algorithm has low sensitivities for the current, former, and temporality unknown smoker groups, the sensitivity for never smokers and notes without smoking status information was high (> 90%), indicating the misclassification was primarily between the three smoking groups. Further follow-up in the form of pre-visit questionnaires, email prompts requesting patients to update their health habits through the EHR web portal, or assessment of smoking status from the patient’s entire record instead of a single note could be utilized to fully establish the patient’s smoking status. Despite this weakness, all patients in the three smoker categories are eligible for inclusion in a Smokers’ Registry. Reducing the misclassification between these groups will improve downstream applications of the registry for identification of patients’ eligible specific services such as cessation counseling or lung cancer screening. Further work on methods for verifying and reconciling smoking status among former and current smokers is needed, but beyond the scope of this paper.

Strengths of this project include the usage of two large cohort of annotated notes to demonstrate both an internal consistency when trained and tested in each as well as the potential for training within one system for application to other systems. This is, to our knowledge, also the first comprehensive NLP-based Smoker’s Registry design that incorporates tools for capturing pack years and quit date as well as smoking status. While we chose to focus exclusively on smoking behavior information (status, pack years, and quit date), annotations for other smoking related concepts were completed by our annotator when curating the local set, which could lead to the development of additional algorithms that pull information relating to smoking attributable diseases and quit attempts in the future.

There are several limitations to the proposed pipeline. While we can accurately predict the presence of information relating to pack years and quit date, the extraction methods we tested for pack years need improvement since approximately 25% of the extracted pack years were actually packs per day or years smoked, but mistakenly identified as complete pack years. Additional rule sets and more advanced algorithms, such as deep neural networks, need to be developed and tested both within our EHR and in external EHRs before these numbers can be utilized for clinical application without manual review of the text window from which the numbers are extracted. Additionally, the cessation date regular expression rules were robust against standard date forms but did not work with non-standard notation or when presented with a date range (example: “patient quit in the late 1980s”), resulting in our low sensitivity in the test set. However, we believe this is also a strength of the algorithm, as the dates reported can be considered unambiguous. We also do not have external notes to be able to fully test our pack year and cessation date rules, as the i2b2 data is not annotated for these concepts, requiring further assessment within external EHRs in the future. Our assessment of these algorithms in the i2b2 notes was useful for showing the potential of this pipeline but cannot be considered a definitive finding. Additional concerns relating to the accuracy of reporting within the EHR need to be evaluated as well due to both issues with patient reporting [48] and a lack of standardized training for information collection on the part of clinicians. Finally, the notes we used to build and test these algorithms are from a teaching and research hospital, which may have systematic differences in reporting from other care centers [49].

Future work includes comparing the data captured by these methods against semi-structured data for visit dates where both are present in the note. If there is a high level of concordance, the proposed pipeline could be used to fill in ‘missing data’ for notes that do not have semi-structured pack year history or cessation dates, reducing the computational burden of the proposed Smoker’s Registry. Additional work comparing our algorithm’s findings against externally recorded smoking data would be useful for assessing the population benefit of a Smokers’ Registry, as it could inform researchers and clinicians about 1) biases in self-reported smoking behaviors to clinicians, 2) clinical recording of smoking behaviors, and 3) the reliability of these tools. This assessment would also allow us to test what proportion of high-risk patients can be identified from their EHR notes for referral to appropriate health services such as cessation counseling or lung cancer screening, and how many individuals would be missed even with this pipeline in place. A long term goal is to either release our completed pipeline through OHDSI [50] or as an Epic app, which will allow for multi-institute adoption of the proposed pipeline. As an intermediate step, we have made our code available through a public Bitbucket repository [51].

## Conclusions

In this paper, we have presented an informatics pipeline for creating a Smokers’ Registry within an EHR, demonstrated that our smoking status algorithm is generalizable, and outlined the future work necessary to complete the proposed registry. Our pipeline is, to our knowledge, the first to attempt to capture from free text documents all the information necessary to determine patient eligibility for lung cancer screening and other testing that is specific to smoking behaviors [24–28]. Improving the synthesis of clinically actionable information using informatics pipelines for clinician and administrative use could lead to better information collection, and ultimately better patient health outcomes. Additional input from policy makers and other clinicians to improve the design of the registry for maximal benefit with minimal time and financial cost will be critical.

## Additional files

Additional file 1: **Table S1.** Stem-word used in our SVM algorithm for hotspot selection in text. This table outlines the specific words utilized to identify relevant text passages usage in the SVM classifier. **Table S2.** Regular expression word conversions and patterns used to collect windows containing smoking behavior information. This table provides the exact regular expression patterns utilized to identify various aspects of smoking history behavior from the free-text notes. **Table S3.** Preliminary Registry Design Including all Smoking Behaviors Information. This table demonstrates one of the potential deliverables of using these algorithms to identify patients in an EHR – a de-identified table reporting on the complete smoking behaviors of the patient population, usable by both clinicians and researchers for population assessment. This information is segregated from patient identifiers to allow for expedited IRB approval or even IRB exemption depending on institutional/governing bodies policies. **Table S4.** Demographics Information Extracted for Administrative and Research Purposes. This table demonstrates another potential deliverable of these algorithms. Specifically, this table has patient identifiers for doing more in-depth patient characteristic studies and for potential re-contact for tobacco related studies. Access to this part of the registry would require full IRB approval for usage in research. The EHR could be modified to provide this list to requesting clinicians for patients they will see that day. (XLSX 14 kb)

Additional file 2: This file contains the code utilized to generate the reported results. The i2b2 results can be validated by anyone. To do so, complete the i2b2’s data user agreement to download the data (or check if your institution already has access) and follow the file set-up outlined in comments in the code. If you encounter any difficulties, please email the first author at ellelpalmer2018@gmail.com for assistance. The Dartmouth-Hitchcock results can be validated at the hospital by any researcher upon approval by our IRB. This can be done using the same script, with the i2b2 load lines commented out and the Dartmouth-Hitchcock lines uncommented. This code file is also available through Bitbucket and will be updated with future works. (PY 36 kb)

## Data Availability

The i2b2 dataset is deidentified and publicly available through i2b2 (https://www.i2b2.org/NLP/DataSets/Main.php) upon completion of a data user agreement by each researcher requesting access. The EHR dataset curated from Dartmouth-Hitchcock records is not deidentified and cannot be released due to HIPAA regulations. However, this data is available for on-site review and usage after IRB approval (contact corresponding author for additional details / arrangement of approval). All code used in this pipeline is available both on Bitbucket (https://bitbucket.org/ellelnutter/tobbacco_user_registery_network_public/) and as Additional file 2. Please note the Bitbucket version is subject to change as this project is ongoing.

## Abbreviations

BRFSS: Behavioral Risk Factor Surveillance System
CMS: Centers for Medicare and Medicaid Services
EHR: Electronic Health Record
i2b2: Integrating Biology and the Bedside
NLP: Natural language processing
